# Antimicrobial Potential of Microorganisms Isolated from the Bottom Sediments of Lake Baikal

**DOI:** 10.3390/antibiotics10080927

**Published:** 2021-07-30

**Authors:** Olga Babich, Margarita Shevchenko, Svetlana Ivanova, Valery Pavsky, Maria Zimina, Svetlana Noskova, Veronika Anohova, Evgeny Chupakhin, Stanislav Sukhikh

**Affiliations:** 1 Institute of Living Systems, Immanuel Kant Baltic Federal University, A. Nevskogo Street 14, 236016 Kaliningrad, Russia; olich.43@mail.ru (O.B.); lionsorciere@gmail.com (M.S.); mariia.zimina@list.ru (M.Z.); svykrum@mail.ru (S.N.); anohovaveronika@yandex.ru (V.A.); chupakhinevgen@gmail.com (E.C.); stas-asp@mail.ru (S.S.); 2 Natural Nutraceutical Biotesting Laboratory, Kemerovo State University, Krasnaya Street 6, 650043 Kemerovo, Russia; 3 Department of General Mathematics and Informatics, Kemerovo State University, Krasnaya Street, 6, 650043 Kemerovo, Russia; pavva46@mail.ru

**Keywords:** extremophilic microorganisms, Lake Baikal, bottom sediments, genetic identification, metabolites, antimicrobial activity

## Abstract

Extremophilic microorganisms attract researchers by their unique characteristics, primarily antagonistic ones, acquired in the process of survival in extreme natural conditions. The antimicrobial potential of the metabolites of these microorganisms is quite broad, from the food industry to therapeutic drugs. Microbial mats of Lake Baikal are a source of unique and diverse microorganisms. The study aimed to evaluate the antimicrobial activity of bacterial strains isolated from the bottom sediments of the lake. Using heterotrophic growth conditions, seven bacterial strains were isolated from samples collected in several coastal zones of Lake Baikal. Thisstudy identified both widespread strains of the genera *Pseudomonas* and *Bacillus* and rare genera *Micrococcus* and *Acinetobacterrepresentatives*. Metabolites of five strains were found to have a broad spectrum of antimicrobial activity. Four large fractions of metabolites of the isolated strains wereidentified. Two peptides of the isolated fractions of metabolites (one is produced by microorganisms of all five isolated strains, another—only by *Pseudomonas putida*) are low molecular weight oligopeptides. These peptides were proved to be bacteriocins.

## 1. Introduction

Lake Baikal covers more than 500,000 km^2^, has up to 500 tributaries (the main ones are the Selenga, Upper Angara, Barguzin, etc.) and a single source (the Angara River) [1]. There are also many thermal springs along the coast of the lake. Each of these water sources makes its adjustments not only for the water balance but also for the diversity of microorganisms in the aquatic world [2,3].

Thermal springs were not considered, since initially, the extremophilic microorganisms with pronounced psychrophilic properties were of interest. Rivers flowing into or out of Lake Baikal can significantly change the species composition of not only the bottom sediments themselves but also the microorganisms inhabiting them. Slyudyanka and Kultuk are located on the coast of one of the bays, in the coastal zone between them several rivers flow into the lake (Slyudyanka, Pokhabikha, Talaya, Kultuchnaya, Medlyanka). Listvyanka is located on the right side of the source of the Angara River (Listvenichny Bay), in its vicinity several streams and rivers flow into Baikal; the largest of them is Krestovka.

Lake Baikal is in the inland rift zone in the south of central Siberia. It is the deepest lake in the world, containing the largest volume of liquid fresh water in one body of water—23,000 km^3^. Morphologically, this lake consists of three deep basins separated by underwater mountain ranges. The southern and central basins have the deepest points (1461 and 1642 m, respectively). The presence of oxygen at the greatest depths, a feature inherent in oceans but unique among deep lakes (>800 m), explains the presence of multicellular life and the evolution of a vast, primarily endemic fauna down to the deepest parts of the lake. The time for complete water substitution in Lake Baikal is approaching 400 years, which is a considerable period for a lake and resembles oceanic reservoirs [4].

The water of Lake Baikal is soft with high hydrocarbonate content. On average, calcium and magnesium bicarbonates account for 84%, chlorides and sulfates 7%, and alkali metals 9% equivalent of ions. Water entering the lake undergoes profound changes in its chemical composition during metamorphization, as a result of which the water of Lake Baikal belongs to the hydrocarbonate–calcium–sulfate (HCO^−3^–Ca^2+^–SO^42+^) hydrochemical facies. It has a faintly alkaline reaction due to the presence of sodium, calcium, magnesium, potassium, and low content of free carbon dioxide. pH is within 7.0–8.5 units and decreases with depth. Additionally, there is a shift towards a neutral reaction (pH 7.0) in winter and alkaline in summer (pH 8.5). In the surface layers of bottom sediments of the lake to a depth of 5–20 cm, there are oxidative processes (iron hydroxyls) and deeper reduction processes (iron sulfates, pyrite). Baikal water contains about 40 chemical elements (in very small quantities); prevalent ones are calcium, carbon, oxygen, magnesium, sodium, potassium, silicon, sulfur, chlorine, nitrogen, iron, phosphorus; meanwhile, iodine deficiency is noticeable. The water has aggressivity and high dissolving power, which increases with depth [5].

The water in Lake Baikal is cold—the temperature of the surface layers does not exceed +8–9 °C even in summer, and in some bays and shores +15 °C (the maximum recorded temperature is +23 °C). The temperature of the deep layers is about +4 °C.

The waters of Lake Baikal are weakly mineralized; the total concentration of dissolved salts is about 0.1 g/L, which is in sharp contrast to the oceans containing approx. 35 g/L.

Lake Baikal is also oligotrophic, and nutrients in the surface layers are recycled about four times before settling into deep waters. All these features, together with a large distance to the nearest sea waters (about 1800 km to the Sea of Okhotsk, northwest of the Pacific Ocean), make Lake Baikal a paradigm of a continental freshwater body, in many ways similar to the ocean, except for sharply reduced salinity. However, this is the only oxygen system comparable to the salty ocean waters in terms of extreme oligotrophy and great depths. Other oxygen lakes, such as Crater Lake (USA), are <1000 m deep. On the other hand, lakes with depths > 1000 m, such as Tanganyika (African lakes), are anoxic. However, only recently Lake Baikal has been used as a model to study differences with the ocean and study the supposed transitions in the microbial community [5]. Since it is generally accepted that life appeared in salty sea waters, which is confirmed by the vital role of monovalent sodium and potassium cations in all living cells, Baikal is an ideal testing ground for studying the adaptation of microbes to life in mineral salt (especially in the absence of sodium) conditions. Distinctive features of the hydrology and hydrochemistry of Lake Baikal (namely its great depth, long period of ice cover, low surface water temperatures even in summer, stable temperatures at the bottom, high oxygen concentrations at all depths, and low concentrations of nutrients) made this lake a very peculiar environment for its inhabitants, including microbial communities [6].

The microbiome of water bodies with a special macrocosm, on which the human impact had minimal consequences, is actively drawing research attention. This includes oceans with their volumes and depths, seas with significant salinity, and Arctic lakes with low temperatures. The living conditions of extremophilic microorganisms endow them with unique properties and the potential for producing unique microbial metabolites [7,8,9,10]. Various aquatic microorganisms are producers of a wide range of compounds with unique properties: antimicrobial, antiviral, anti-inflammatory, and other activities [11,12].

Lake Baikal is one of these water bodies: the largest natural source of freshwater, low temperature of water layers, the presence of unique fauna representatives, including individual microorganisms, found only in this lake [13,14]. The total microorganism number in the bottom sediments of Lake Baikal reaches several billion cells per gram of wet soil (maximum 3.5 billion cells/g), which varies depending on the litho logical composition and the place of sediment collection: the number is greater in silts than in sands; more in shallow-water sediments than in deep-water ones. Bacteria of various physiological groups participating in the carbon, nitrogen, and phosphorus cycles were found in the water and sediments of Lake Baikal. The Lake Baikal microbiome is being actively studied—the volume of obtained information is constantly growing.

The structure and functional diversity of the microbial community of mineral springs largely depend on the chemical composition of waters and sediment. The content of electron acceptors, biogenic elements, and substances in mineral water affects the formation of the composition of the psychrophilic microbial community and the potential of its antimicrobial metabolism [15].

Close trophic relationships between various groups of psychrophilic microorganisms allow them to effectively participate in the transformation of organic and inorganic substances due to their enormous biochemical and microbiological potential [16].

Microbial communities in Lake Baikal can have significant biomass and form so-called microbial mats. Representatives of different trophic groups of microorganisms, carrying out the full cycle of biogenic elements, interact in them. Microbial communities of Lake Baikal can be divided into two types: dominated by phototrophic microorganisms and dominated by chemotrophic microorganisms. The chemotrophic communities of Lake Baikal often develop in the form of fouling. The border between phototrophic and chemotrophic communities is determined by the resistance of the photosynthetic apparatus to environmental factors, primarily to temperature. Phototrophic microorganisms play a crucial part in the formation of the community. At present, cyanobacterial mats are found mainly in biotopes where extreme conditions have been preserved, as in Lake Baikal [17].

This study aimed to screen extremophilic microorganisms isolated from the bottom sediments of Lake Baikal and characterize antagonistic properties of their metabolites as primary substances for antimicrobial drugs.

## 2. Results

Seven isolates, differing in morphological characteristics (Appendix A), were isolated from the bottom sediments sampled near these places. The bacteria were classified based on the microscopic morphological description of the colony of culture characteristics, physiological characteristics, and molecular typing by the RAPD method. Six isolates were rod-shaped bacteria, and one isolate was spherical bacteria (cocci). Among rod-shaped bacteria, there are both Gram-positive (isolate No. 1) and Gram-negative (isolates No. 2–4, No. 6, No. 7) forms.

The simplicity of the external form of microorganisms and the structure of their cells made classification by morphological characteristics difficult. In this regard, additional information on the species of microorganisms isolated from the bottom sediments of Lake Baikal was obtained by 16S rRNA gene sequencing. Phylogenetic trees are presented in Figures S1–S7.

16S rRNA gene sequencing (Appendix B, Appendix C, Appendix D, Appendix E, Appendix F, Appendix G and Appendix H) showed that the isolates belong to the following species: isolate 1 *Bacillus megaterium*, isolate 2 *Pseudomonas fluorescens*, isolate 3 *Pseudomonas putida*, isolate 4 *Pseudomonas aeruginosa*, isolate 5 *Micrococcus luteus*, isolate 6 *Pseudomonas oleovorans*, isolate 7 *Acinetobacter calcoaceticus*.

Although flora and fauna of the lake have long attracted the attention of scientists, the microcosm has not been studied enough. Many works are devoted to bioplankton [14,15], and the study of bacterial communities began relatively recently. Phylogenetic analysis of microbial genes of 16s rDNA showed that actinomycetes account for up to 30% of the microorganisms present in the lake, most of which are micromonospora and streptomycetes. Of particular interest are clusters of microorganisms in the places of hydrocarbon and petroleum products. They can destroy them and expand the possibilities of preserving the eco-world, both in this lake and in other water bodies with significant anthropogenic impact [16,17,18,19,20]. It is established that among the oil-oxidizing microorganisms in the bottom sediments, the bacteria of the genus *Bacillus* dominated, while in the water column—*Rhodococcus*, *Pseudomonas*, and *Micrococcus*.

Attempts to identify the best conditions for growing in a flask at various temperatures (4 °C, 10 °C, 15 °C, 20 °C, 25 °C, 30 °C, and 37 °C) of bacteria isolated from Lake Baikal showed that most of them grow more abundantly in low-concentration environments in the temperature range of 30 to 37 °C, indicating that all these isolates are psychrotrophic. Metabolites produced by these microorganisms under extreme conditions, among other things, are the reaction of these organisms to adaptation. Therefore, the characteristics of extremophilic microorganisms isolated from the bottom sediments of Lake Baikal determine their potential for new antimicrobial drugs; their antibacterial effect on microorganisms pathogenic for humans was studied. *Salmonella enterica* (causes agent of diseases such as typhoid fever, paratyphoid fever, salmonellosis), *Escherichia coli* (gastroenteritis, inflammation of the genitourinary system, meningitis in newborns), *Bacillus cereus* (causes foodborne toxicoinfections in humans, produces enterotoxins), *Enterococcus faecalis* (causes intra-abdominal infections, septicemia, and meningitis in humans), *Candida albicans* (causes human opportunistic infections transmitted through the mouth and genitals), *Staphylococcus aureus* (pneumonia, meningitis, osteomyelitis, endocarditis, infectious toxic shock, sepsis, etc.) were selected as testcultures.

Antimicrobial activity was determined by the diameter of the lysis zone (microbial growth inhibition zone) exposed to the action of peptides. The larger the lysis zone, the higher the antimicrobial activity, because pathogenic and opportunistic microorganisms are suppressed over a larger area of action of peptides. The antimicrobial properties of isolated strains were assessed by two methods. The results of evaluating the antimicrobial properties of microorganisms are presented in Table 1. Five isolated microorganisms No.1–4, 6 (*Bacillus megaterium*, *Pseudomonas fluorescens*, *Pseudomonas putida*, *Pseudomonas aeruginosa*, *Pseudomonas oleovorans*) have antimicrobial properties against all tested strains. The antagonistic activity of the supernatant of cell-free cultures of these five strains ranged from 11.0 ± 0.1 to 21.0 ± 0.4 mm in the first method and 25.1 ± 0.6 to 38.2 ± 1.2 mm in the second method for determining the antimicrobial activity. Strain No. 4 showed a strong antibacterial activity from 16.1 ± 0.2 mm against the *S. enterica* strain to 21.0 ± 0.4 against *E. coli* in the first method. In the second method, strain No. 3 (*E.coli,* 38.2 ± 1.2 mm; *B.cereus*, 36.4 ± 1.2 mm) and strain No. 6 (*S. enterica*, 36.1 ± 0.5 mm; *E. coli*, 38.2 ± 1.6 mm; *C. albicans*, 37.0 ± 1.8 mm) showed the highest activity. The broadest antimicrobial activity spectrum among the strains was observed in strains No. 2 and No. 6. Two microorganisms, No. 5 and No. 7 (*M. luteus* and *A. calcoaceticus*), did not show antimicrobial activity against any of the test cultures and were removed from subsequent stages of the study.

Resistance was evaluated by the presence and diameter of the lysis zone (Table 2). The studied strains of microorganisms *B. megaterium* and *P. fluorescens* showed pronounced antibiotic resistance to all four antibiotics, while *P. aeruginosa* and *P. oleovorans* were sensitive to ampicillin, *P. putida*—to kanamycin.

**Figure 1:**
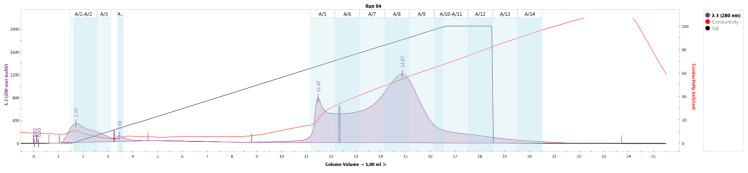
The identification of metabolites produced by selected microorganisms (*B. megaterium*, *P. fluorescens*, *P. putida*, *P. aeruginosa*, *P. oleovorans*).

Studies of metabolites produced by selected psychrotrophic microorganisms (*B. megaterium*, *P. fluorescens*, *P. putida*, *P. aeruginosa*, *P. oleovorans*) (Table 3, Figure 1) resulted in the identification of four large fractions, A/1–A/4, A5/–A/7, A/8–A/11, A/12–A/14, presumably containing bacteriocins.

**Figure 2:**
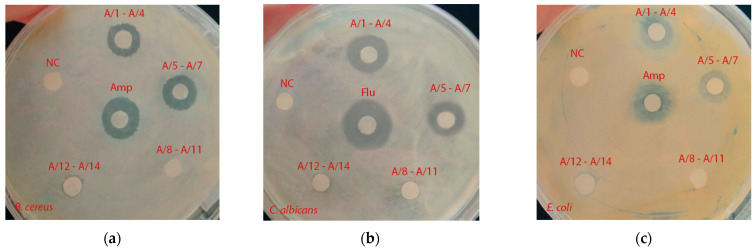
The evaluation of the antimicrobial properties of isolated fractions: (**a**) *B. cereus*; (**b**) *E. coli* and (**c**) *C. albicans*. Amp—Ampicillin; Flu—Fluconazole; NC—sterile environment.

The antibacterial activity of isolated fractions against *E. coli*, *B. cereus* and *C. albicans* was investigated (Table 4, Figure 2).

**Figure 3:**
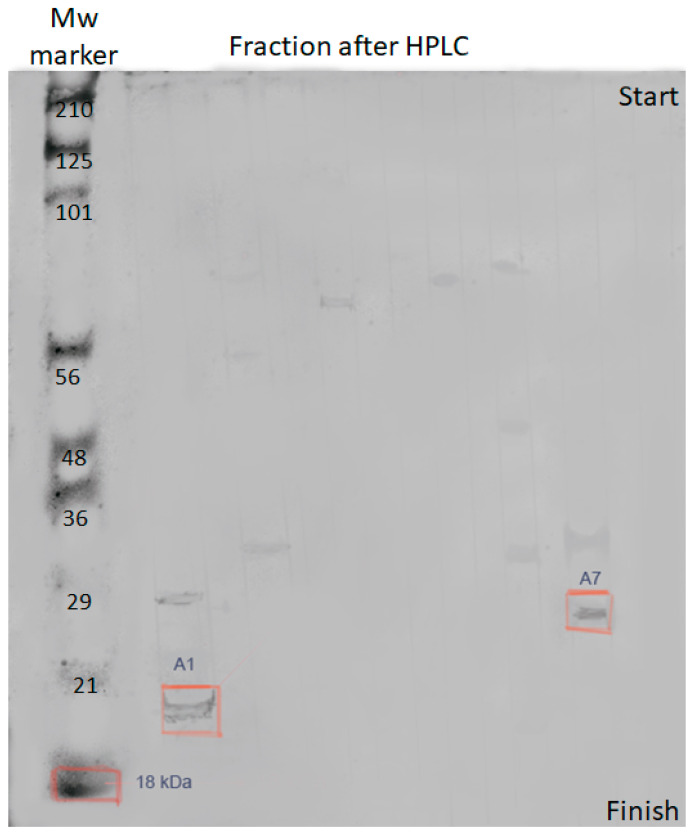
PAGE of fractions of metabolites A/1 and A/7. PAGE of individual fractions isolated y HPLC (peptides for fractions A1 and A7 are indicated by colored squares), weighing from 29 to 18 kDa, for which the sequence was established by the MALDI-TOF method.

The antibacterial activity was found in fractions A/1 and A/7. Polyacrylamide gel electrophoresis (Figure 3) was used to confirm that the detected metabolites are bacteriocins.

**Figure 4:**
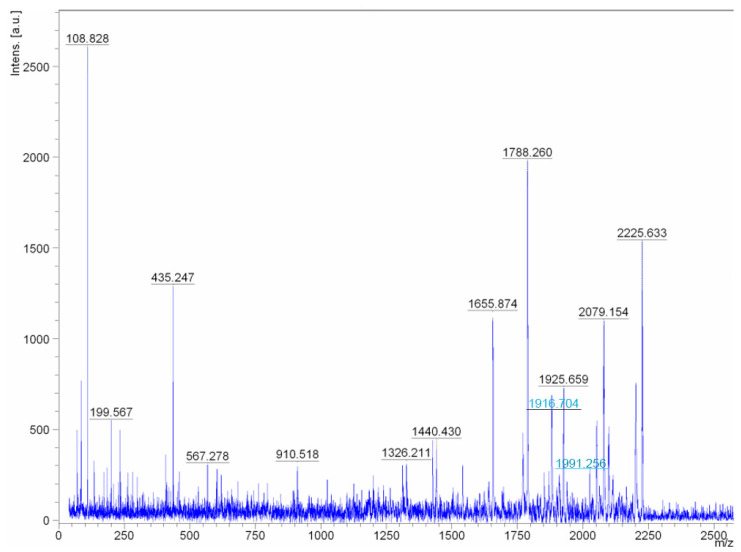
Molecular fragments of peptides of fractions A/1 obtained by the MALDI-TOF/TOF method.

**Figure 5:**
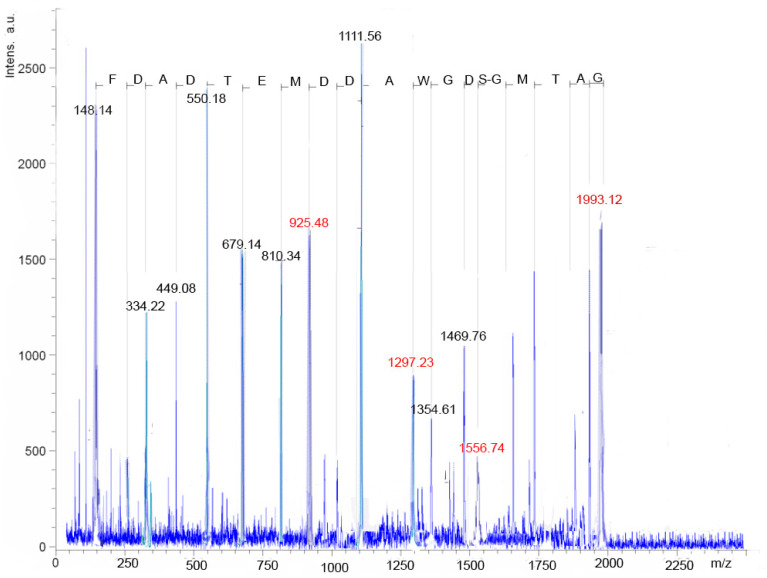
Amino acid annotation for molecular ion fragments in LIFT MALDI-TOF/TOF (MS/MS) experiments.

It was found that the studied compounds are low molecular weight oligopeptides (Figure 4 and Figure 5). The results showed that the metabolites of fractions A/1, A/7are active at neutral and low alkaline pH. The peptides lose their activity at temperatures above 45 °C.

**Figure 6:**
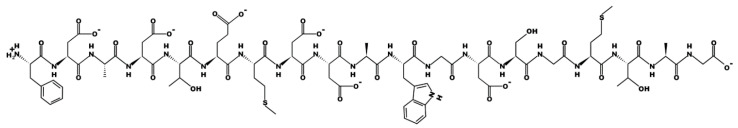
Graphic representation of the amino acid sequence of metabolite fragments of fractions A/1.

The structure of peptides, revealed by chromatography, may be due to a peptide bond between the amino group of the N-terminal amino acid and the carboxyl group of the C-terminal amino acid. The fragmentation of the peptide of fractions A/1, A/7 is shown in Figure 6, Appendix I. It was found that peptides of fractions A/1, A/7 have a similar structure. The peptide of fraction A/1 was found only in the culture liquid of *Pseudomonas putida*.

Reanalysis of the spectra of two ions with masses 1916.704 and 1991.256 (the masses are highlighted in Figure 4 as peaks with blue inscriptions) allowed concluding that these are not different peptides, but one molecular ion of a peptide with and without a C-terminal amino acid. The peptide fragment sequence exhibiting biocidal activity in fractions A/1 and A/7 is the following: PhenAspAlaAspThrGlMetAspAspAlaTrpGlyAspSerGlyMetThrAlaGly.

## 3. Discussion

*Pseudomonas* bacteria have the highest antimicrobial potential among the obtained isolates. The *M. luteus* strain was identified among the isolates of Arctic lakes [21]. It did not show antimicrobial activity against *S. aureus*, *E. faecium*, *E. coli*; *P. aeruginosa*, *C. albicans*, *A. fumigatus*, *C. neoformans*.

Our data confirm that microorganisms of Lake Baikal can produce biologically active molecules [22] endowed with a new structure and mechanism of action. Similar studies were carried out in Antarctic lakes, in which significant groups of microorganisms with antimicrobial and antifungal properties were isolated [4,6,21,23,24].

Even though flora and fauna of lakes have long attracted the attention of scientists, the microcosm has not been sufficiently studied. There are many studies on bioplankton [14,15], and the study of bacterial communities began relatively recently. Phylogenetic analysis of 16s rRNA microbial genes showed that actinomycetes account for up to 30% of microorganisms found in the lake, most of which are *micromonospora* and *streptomycetes*. The accumulations of microorganisms in the places where hydrocarbons and oil products emerge are of particular interest. They can destroy and expand the possibilities of preserving the eco-world, both in this lake and in other reservoirs with significant anthropogenic impact [16,17,18,19,20]. It was found that among the oil-oxidizing microorganisms of bottom sediments, bacteria of the genus *Bacillus* dominate, and in the water—*Rhodococcus*, *Pseudomonas*, and *Micrococcus*.

In our isolates, bacteria of the genus *Pseudomonas* predominated; similar results were established by many authors [16,25] who studied Baikal. Most of the microbial community of the water and bottom sediments of Lake Baikal are non-spore-forming rods. Representatives of 7 phylogenetic groups of the genus *Pseudomonas* were found in the water and bottom sediments. The number of these microorganisms in the water of Northern Baikal is 21% of the number of cultivated heterotrophs, in the Middle—22–48%, in the South—35–38%. It has been established that natural *Pseudomonas* strains isolated from the water and bottom sediments of Lake Baikal are active producers of exoenzymes, due to which the process of biodegradation of both simple and complex high-molecular compounds occurs, which contributes to the preservation of the purity of Baikal waters. The authors [12,25] studied the microbial community in bottom sediments and soils of the floodplain and on the islands of the Selenga delta, respectively. The fungi population in the studied soils was low, and *Bacillus, Pseudomonas*, and *Aquaspirillum* predominated among the bacteria. *Streptomycetes* constituted a significant part of the microbial complexes of floodplain soils, while in the soils of the islands, their number was insignificant. 57 strains of the genus *Pseudomonas* were isolated from the bottom sediments of the Selenga delta and actinomycetes, representatives of the genus *streptomycetes* and *micromonospora*, were found there as well. In the bottom sediments of the near-delta area of the Selenga River, the content of bacteria of the genus *Pseudomonas* ranges from 18% to 47%, in sediments from the delta border—from 62% to 88%, in the area of the cellulose plant—does not exceed 20% of the total number of heterotrophs. Therefore, bacteria of the genus *Pseudomonas* dominate mainly in the lake water areas that are not influenced by anthropogenic factors. The Selenga River influences the formation of the microbial community in the bottom sediments of the study area. Most studies expand information on the species representation of microorganisms and their cultivation conditions without examining their antimicrobial potential.

## 4. Materials and Methods

### 4.1. Sediment Sampling

**Figure 7:**
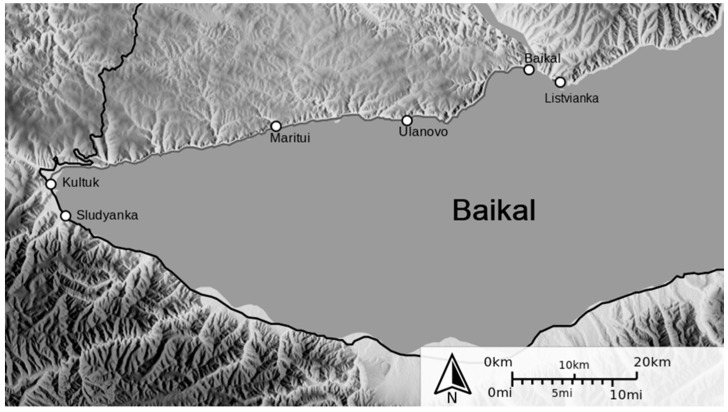
Sediment sampling sites.

The material for the isolation of pure cultures of extremophilic (psychrotrophic) microorganisms was the bottom sediments of Lake Baikal, sampled in the coastal zone (Slyudyanka, Listvyanka, Kultuk, Figure 7) in May–June 2018.

Seven isolates were isolated from 9 samples of bottom sediments collected in the coastal zone of Lake Baikal (Slyudyanka 51°40′00″ N; 103°42′00″ E; Kultuk 51°41′20″ N; 103°47′00″ E; Listvyanka 51°51′11″ N; 104°52′55″ E; Western end, Figure 1) in May–June 2018.The temperature of all bottom sediment samples during sampling was 4.0 ± 0.5°. The samples were collected near the coast at a depth of 2–3 m, only the upper layer of bottom sediments was removed [4,24,26]. Selected samples were placed in sterile Petri dishes and frozen at a temperature of −20 °C; after returning to the laboratory, they were stored at −80 °C until further analysis. To isolate colonies of microorganisms, direct inoculation on solid nutrient media was performed by the surface method.

### 4.2. Production of Microorganism Cultures

Inoculation material was used in the form of soil.To isolate colonies of microorganisms, samples of bottom sediments were crushed under sterile conditions, and a small piece (~5 g) was spread on the surface of a plate with an agar nutrient medium. The plates and tubes were incubated stationary for 3 days.

### 4.3. Production of Pure Isolates

Pure cultures were grown in liquid nutrient media, in 5 mL test tubes stationary for 3 days at temperatures of 4 °C, 10 °C, 15 °C, 20 °C, 25 °C, 30 °C, and 37 °C to establish which bacteria dominated at temperatures between 10 and 20 °C. At the same time, strict (obligate) psychrophiles cannot reproduce at temperatures above 20 °C, and facultative (also called psychrotrophs) have an optimum growth of 22 to 30 °C. This way, bacteria were classified either as facultative psychrophile.

In the identification process, isolation and cultivation were carried out on a nutrient medium for bacteria of the genus *Bacillus* (peptic digest of animal tissue 10.0 g/L, beef infusion 500.0 g/L, sodium chloride 5.0 g/L, agar–agar 25.0 g/L); genus *Pseudomonas* (peptone 10.0 g/L, yeast extract 5.0 g/L, sodium chloride 10.0 g/L, K_2_HPO_4_ 1.5 g/L); the genus *Micrococcus* (casein hydrolyzate 1.8 g/L, yeast extract 2.7 g/L, sodium chloride 0.2 g/L); genus *Acinetobacter* (pancreatic digest of casein 1.0 g/L, pepsin digest of meat 0.5 g/L, yeast extract 1.0 g/L, starch 0.5 g/L, beef infusion 5.0 g/L).

### 4.4. Identification of Isolates

To detect Gram staining of bacteria using a Gram staining kit (Lab—Biomed, Moscow) and API—50 test systems (Promix, Novosibirsk, Russia), direct microscopy (biological microscope AxioScopeA1, Carl Zeiss, Oberkochen, Germany) and mobility tests were used. For microscopy, a drop of culture was taken with a loop or Pasteur pipette and, placing the drop in the middle of dry clean glass, and it was evenly distributed in the form of a stroke. The strokes were dried at room temperature in the air and fixed. To study the micro- and macromorphological features of bacteria, they were stained and studied for size, shape, location, etc. [27]. Each recognizable colony type was streaked onto fresh medium to obtain pure cultures. Fresh medium was prepared immediately before obtaining pure cultures and consisted of liquid culture media corresponding to the species of microorganisms after identification. Cell sizes were determined under a microscope using an eyepiece micrometer. Bacterial motility was determined by microscopic examination of cultures using agar medium with mobility using the hanging drop method.

Purified colonies of various types were identified to the genus or species level using standard molecular biology tests according to the criteria given in Burgey’s Manual of Systematic Bacteriology (https://www.bergeys.org/publications/#archea-bacteria (accessed on 1 June 2021)). The criteria are based on identification using polymerase chain reactions and phylogenetic analysis using the MIKROLATEST test systems. MICROLATEST^®^ Kits are 96-well microtiter plates with 1, 2, or 3-row vertical strips for setting 8, 16, or 24 biochemical reactions. The wells contained dehydrated substrates. When a suspension of microorganisms was added, the substrates dissolved; during incubation, biochemical reactions took place, the results of which were recorded by the change in the indicator color after adding the reagent automatically using a Multiscan photometer. Identification was based on a study of the nucleotide sequences of 16S rDNA. This panbacterial gene is present in all prokaryotes; the determination of its primary structure allowed studying the homology degree of microorganisms and their evolutionary relationships. In accordance with the analysis of the structure of nucleotide sequences of 16S rDNA, type strains, species, and genus of microorganisms were identified. Bergey’s guide includes name-ids—NamesforLife (N4Lids). This allowed identifying microorganisms, as well as related concepts, names, and terms in real time through the available Internet resources.

### 4.5. Isolation of Metabolites

Metabolites were produced as follows: pure cultures of microorganisms were grown in liquid nutrient media corresponding to the species of bacteria. Then, suspensions of microorganisms were obtained, centrifuged, and the supernatant was filtered through 22 µm membrane filters. The resulting sterile solution of metabolites was used for experiments [28].

### 4.6. Determination of the Taxonomic Affiliation of Isolates

The taxonomic affiliation of the isolates was determined by sequencing the 16S rRNA gene. DNA was isolated by phenol-chloroform extraction. The complete 16S rRNA gene sequence was amplified using primers 27F 5′-AGAGTTTGATCCTGGCTCAG-3′; 1492R 5′-GGTTACCTTGTTACGACTT-3′on C1000 Touch Thermal Cycler (Bio-Rad, Hercules, CA, USA). Cycling parameters: denaturation—95 °C, 15 s; annealing of primers—57 °C, 30 s; elognation—72 °C, 1 min; the number of cycles—30. To obtain amplicons, we used a ready-made Encyclo Plus PCR Kit (Evrogen, Moscow, Russia), which provides the ability to clone products into T-vectors. The reaction products were purified by horizontal 1% agarose gel electrophoresis using the CleanUp Mini kit (Evrogen, Russia).

The resulting amplicons were used to obtain libraries for subsequent capillary sequencing. The 16S rRNA gene sequences were cloned into the pAL2-T vector using the Quick-TA kit (Evrogen, Russia). Recombinant vectors were used to transform chemically competent *E. coli* DH5alfa cells by heat shock. Transformants were placed on Petri dishes with a dense nutrient medium LB-agar (tryptone—1%, agar—1.5%, yeast extract—0.5%, NaCl—1.0%). Ampicillin at a final concentration of 50 mg/L and IPTG/X-gal were used as selective markers. Petri dishes with transformants were cultured for 8–10 h at 37 °C. The presence of an insert was determined by a blue–white test and additional PCR screening using primers M13 Forward (5′-GTTGTAAAACGACGGCCAGTG-3′) and M13 Reverse (5′-AGCGGATAACAATTTCACACAGGA-3′). *E. coli* colonies showing the presence of a recombinant vector with an insert of the 6S rRNA gene were placed in 5 mL of liquid LB culture medium and cultured to an optical density OD_600_ equal to 0.6–0.8.Recombinant vectors were isolated from liquid culture using the Plasmid Miniprep kit (Evrogen, Moscow, Russia).

The sequencing reaction was set up in 10 μL using purified vectors, primers M13 Forward (5′-GTTGTAAAACGACGGCCAGTG-3′) and M13 Reverse (5′-AGCGGATAACAATTTCACACAGGA-3′), and BigDye Terminator v3.1 Cycle Sequencing Kit (Thermo Scientific, Waltham, MA, USA). Libraries were purified using BigDyeXTerminator™ Purification Kit (Thermo Scientific, Waltham, MA, USA). Capillary sequencing was performed on an Applied Biosystems 3730 DNA Analyzer (Applied Biosystems, Foster, KA, USA).

Bioinformatic processing, including trimming of the 5′ and 3′ ends and assembly of sequences, was carried out using the CLC Genomics Workbench 8.5 program. The resulting assembly of 16S rRNA genes for each sample was aligned against the bacteria and archaea database using the Standard Nucleotide BLAST tool.Based on the BLAST results, phylogenetic trees were constructed using the MEGA software package.

### 4.7. Assay for Antimicrobial Substance Production

*Salmonella enterica* ATCC 14028, *Escherichia coli* B-6954, *Bacillus cereus* EMTC 1949, *Alcaligenes faecalis* B1820, *Candida albicans* EMTC 34, *Staphylococcus aureus* ATCC 25923, were used as test strains, which were taken from the laboratory collection of the Research Institute of Biotechnology of Kemerovo State University.

Test cultures were cultivated overnight in specialized media at temperatures from 30 °C to 37 °C, depending on the strain, the pH of the medium was 6.8–7.0. Bacteria were cultivated on nutrient media, the composition of which corresponds to commercial *S. enterica*—Rappaport–Vassiliadis medium (Sigma–Aldrich, St. Louis, MO, USA), cultivation temperature 35 °C; *E. coli*—LB (Sigma–Aldrich, St. Louis, MO, USA), cultivation temperature 37 °C; *B. cereus*—Mueller Hinton Broth (Sigma–Aldrich, St. Louis, MO, USA), cultivation temperature 37 °C; *S. aureus*—Giolitti–Cantoni broth (Sigma–Aldrich, St. Louis, MO, USA), cultivation temperature 35 °C; A. *faecalis*—LB (Sigma–Aldrich, St. Louis, MO, USA), cultivation temperature 30 °C; *C. albicans*—Sabouraud medium (Sigma–Aldrich, St. Louis, MO, USA), cultivation temperature 30 °C. The obtained suspensions of microorganism cells were used to determine the antimicrobial activity.

The antimicrobial activity of the isolated strains was analyzed using two methods:

1. Antimicrobial activity of the isolated strains was determined using the agar disc diffusion method [29,30,31] under anaerobic conditions. The prepared sterile paper disks were dipped in the culture liquid, and the excess was pressed off. Antimicrobial discs impregnated with metabolites of microorganism strains obtained according to 2.5 were applied to agar with test strains, observing the rules of asepsis (safety rules for working with pathogenic microorganisms that can be isolated along with the bacteria under study must be observed). The disks were positioned so that the distance between their centers was at least 24 ± 4 mm. After placing the discs on agar, they were pressed with a sterile needle or pincers until they fully contact the surface of the medium.

15 ± 5 min after placing the discs, the Petri dishes were inverted and incubated in an aerobic atmosphere at 37 ± 2 °C for 24 h. After incubation, the diameter of the complete incubation zones was measured (according to observation data without microscopy, visually), including the diameter of the disc, to the nearest whole millimeter using a ruler [32,33,34]. Individual colonies of microorganisms, which could hardly be detected at the edge of a noticeable zone of inhibition, were not considered (MUK 4.2.1890-04 Determination of the sensitivity of microorganisms to antibacterial drugs. Methodological Guidelines).

2. Determination of antimicrobial activity using a two-layer nutrient medium. The bottom layer was a solid medium—meat-and-peptone agar or LB.A total of 2 cm lines of microorganisms were made on the nutrient medium in the center of the Petri dish. The cultivation was carried out at a temperature of 37 ± 2 °C and 30 ± 2 °C during 24 h.

At the end of the cultivation, the test cultures were diluted in saline solution. Optical density at 600 nm ranged from 0.008 to 0.013, which corresponded to a cell content of approximately 1.5 × 10^8^ CFU/mL when scaled by the McFarland method. Then saline solution with test cultures was poured into meat-and-peptone agar and poured as a second layer into Petri dishes with grown microorganisms. The cultivation was carried out at a temperature of 37 ± 2 °C and 30 ± 2 °C for 24 h. After the specified time, antimicrobial activity was observed by measuring the diameter of the developed antimicrobial ring.

### 4.8. Antibiotic Resistance and Minimal Inhibitory Concentration

Four antibiotics (ampicillin, tetracycline, kanamycin, chloramphenicol) with proven antibacterial activity against the studied species were chosen to determine the sensitivity of the studied strains. Antibiotic resistance was determined by the disc diffusion method [35].

The strains were cultured on a solid agar nutrient medium and a liquid LB nutrient medium at a temperature of 25 °C. To obtain an inoculum, five colonies of the same type, taken from an overnight culture, were inoculated by the thinning streak method, placed in 5 mL of liquid LB medium, and cultured in a shaker incubator at 25 °C to an OD_600_ of 0.008–0.013, which corresponds to a cell content of approximately 1.5 × 10^8^ CFU/mL. The resulting inoculums in a volume of 1 mL were plated on Petri dishes with LB-agar and distributed by rocking. For 10 min, Petri dishes with inocula were dried at room temperature, after which preprepared sterile paper disks soaked in antibiotic solutions prepared based on 0.9% sodium chloride solution were applied at the rate of 1 mL of solvent per 100,000 antibiotic units. Discs moistened with 0.9% sodium chloride solution were used as negative controls. After 15 min, required for the prediffusion of the antibiotic into agar, the strains under study were incubated for 24 h at a temperature of 25 °C. To determine the minimum inhibitory concentrations of antibiotic-sensitive microorganisms, the experiment was carried out according to a similar scheme using the following dilutions of antibiotics: 500 mg/L, 50 mg/L, 25 mg/L, 10 mg/L, 5 mg/L.

### 4.9. Identification of Metabolites

Selected strains of microorganisms were grown in LB liquid nutrient medium at a temperature of 25 °C. To obtain an inoculum, five colonies of the same type, taken from an overnight culture, were inoculated by the thinning streak method, placed in 5 mL of liquid LB medium, and cultured in a shaker incubator at 25 °C to an OD_600_ of 0.008–0.013, which corresponds to a cell content of approximately 1.5 × 10^8^ CFU/mL. The resulting cell suspensions were centrifuged for 15 min at 5000 rpm. The culture fluid was separated from the cell debris by filtration and lyophilized using a FreeZone freeze dryer (Labconco, Kansas City, MO, USA) at −20 °C. The resulting dry residue was extracted with methanol to remove organic compounds and then dissolved in 10 mL of tris-glycine buffer, pH 3.5. This method of extraction allows isolating the peptide fraction predominantly [36,37].

The resulting solution containing the peptide fraction was subjected to chromatographic separation using an NGC HPLC chromatograph (Bio-Rad, Hercules, CA, USA) on an Uno-Q1 column (Bio-Rad, Hercules, CA, USA) at a gradient pH 2.5–8.9. The eluent was buffer solutions: phase A—citrate–phosphate buffer with pH 2.5; B–tris-glycine buffer pH 8.5; gradient phase B 0–100% for 15 column volumes (1 column volume—1 mL). Chromatography parameters: Run 04, Trace Type: λ 3 (280 nm), Best Fit: 8, Slope: 10, Sensitivity: Medium, Size: N/A.

Electrophoresis using a ready-made PAA gel (Bio-Rad, Hercules, CA, USA) and TGB buffer (Tris-base 25 mM, glycine 250 mM, SDS 0.1%, water) at a voltage of ~10 V/cm in a concentrating gel, and ~180 V in the resolving gel was used to determine if the isolated metabolites are bacteriocins.

The amino acid sequence of metabolites was determined by tandem mass spectrometry MALDI-TOF/TOF. LIFT mode was used to analyze daughter ions resulting from fragmentation of the molecular ion of a biocidal peptide. MALDI-TOF/TOF spectra were obtained under the following conditions: reflector 18 kV, ion source 8 kV, ion gate 12kV, second reflector 24 kV. The Anchor Chip target was inserted into an Autoflex TOF/TOF instrument (Bruker, Billerica, MA, USA) and submitted to an automated analysis loop using external mass calibration. Following MS acquisition each spectrum was submitted to a peptide mass fingerprint search using the ProteinScape proteomics database system (Bruker). After automated assessment of the search results only those samples not unambiguously identified by PMF were automatically submitted to LIFT TOF/TOF acquisition by the workflow control system (WARP, Bruker). A maximum of four precursor ions per sample were chosen for MS/MS analysis via BioTools 2.2 with the RapiDeNovo extension (Bruker).

The pH spectrum, in which the peptides exhibit activity, was determined using cultivation according to the above-described method for determining antibacterial activity by adding hydrochloric acid and sodium hydroxide to the LB medium to change the acidity.

Thermal stability was determined by cultivation in the temperature range from 25 °C to 50 °C.

### 4.10. Statistical Analysis

Each experiment was repeated three times. The data in the tables were expressed as means ± SE. Data processing was carried out by standard methods of mathematical. Homogeneity of the sampling effects was checked using the Student’s *t*-test. Differences between means were considered significant when the confidence interval is smaller than 5% (*p* < 0.05).

## 5. Conclusions

Ancient Lake Baikal is distinguished by significant volumes of natural freshwater. Despite human activities, the lake still maintains a healthy status, primarily due to the diverse microbial communities that inhabit it. For us, Baikal was of interest as a source of microorganisms with extended antimicrobial characteristics, metabolites of which could be used as a base for antimicrobial drugs. Seven strains were isolated from bottom sediment samples, most of which were attributed to bacteria of the genus *Pseudomonas*. Four large fractions of metabolites of the isolated strains were identified. Two peptides of these fractions were proved to be bacteriocins using the method of determining antimicrobial activity. Further research will be focused on determining the safety and potential of the isolated antimicrobial peptides as the base for bactericidal pharmaceutical substances.

## Figures and Tables

**Table 1:** The evaluation of the antimicrobial properties of microorganisms isolated from bottom sediments of Baikal Lake.

Strains	Antimicrobial Activity (Diameter of the Growth Inhibition Zone, mm)					
	I	II	III	IV	V	VI
Disk diffusion method						
No. 1	15.1 ± 0.2	13.4 ± 0.1	11.0 ± 0.1	15.9 ± 0.2	12.1 ± 0.1	15.0 ± 0.0
No. 2	16.3 ± 0.1	18.1 ± 0.3	14.2 ± 0.2	18.8 ± 0.3	17.2 ± 0.3	14.9 ± 0.1
No. 3	13.0 ± 0.2	16.1 ± 0.1	14.1 ± 0.1	16.0 ± 0.1	12.0 ± 0.1	17.1 ± 0.2
No. 4	16.1 ± 0.2	21.0 ± 0.4	18.2 ± 0.2	20.1 ± 0.2	17.1 ± 0.3	19.2 ± 0.2
No. 5	0.0 ± 0.0	0.0 ± 0.0	0.0 ± 0.0	0.0 ± 0.0	0.0 ± 0.0	0.0 ± 0.0
No. 6	15.2 ± 0.2	18.2 ± 0.3	16.1 ± 0.2	14.1 ± 0.2	16.9 ± 0.2	12.7 ± 0.3
No. 7	0.0 ± 0.0	0.0 ± 0.0	0.0 ± 0.0	0.0 ± 0.0	0.0 ± 0.0	0.0 ± 0.0
Two-layer nutrient medium method						
No. 1	27.4 ± 1.0	32.3 ± 1.1	30.0 ± 1.4	25.1 ± 0.6	31.2 ± 1.6	29.1 ± 1.0
No. 2	31.1 ± 0.9	33.1 ± 0.9	26.9 ± 0.7	29.8 ± 0.4	32.1 ± 1.3	31.2 ± 1.0
No. 3	34.1 ± 0.4	38.2 ± 1.2	36.4 ± 1.2	31.7 ± 1.1	32.9 ± 1.1	31.1 ± 1.6
No. 4	29.2 ± 0.6	28.1 ± 0.8	31.1 ± 1.0	26.0 ± 0.9	27.1 ± 0.9	28.0 ± 1.1
No. 5	0.0 ± 0.0	0.0 ± 0.0	0.0 ± 0.0	0.0 ± 0.0	0.0 ± 0.0	0.0 ± 0.0
No. 6	36.1 ± 0.5	38.2 ± 1.6	35.3 ± 1.0	33.8 ± 1.7	37.0 ± 1.8	33.8 ± 0.9
No. 7	0.0 ± 0.0	0.0 ± 0.0	0.0 ± 0.0	0.0 ± 0.0	0.0 ± 0.0	0.0 ± 0.0

I—S. enterica; II—E. coli; III—B. cereus; IV—A. faecalis; V—C. albicans; VI—S. aureus. No. 1—B. megaterium; No. 2—P. fluorescens; No. 3—P. putida; No.4—P. aeruginosa; No. 5—M. luteus; No. 6—P. oleovorans; No. 7—A. calcoaceticus. The data are expressed as mean ± SE (n = 3). The antagonistic activity among different bacterial cell-free culture supernatant was statistically significant (*p* < 0.05), and different test pathogenic bacterial strains was not significant.

**Table 2:** Evaluation of antibiotic resistance (presence of a lysis zone) of microorganisms isolated from bottom sediments of Lake Baikal.

Microorganism	Antibiotic			
	Ampicillin	Tetracycline	Kanamycin	Chloramphenicol
*Bacillus megaterium*	−	−	−	−
*Pseudomonas fluorescens*	−	−	−	−
*Pseudomonas putida*	−	−	+	−
*Pseudomonas aeruginosa*	+	−	−	−
*Pseudomonas oleovorans*	+	−	−	−

“+”—the presence of a lysis zone; “−”—the absence of a lysis zone.

**Table 3:** Results of identification of metabolites produced by selected microorganisms.

№	Rack/Tube	Relative Area (%)
1	A/1	0.37
2	A/2	5.80
3	A/3	1.58
4	A/4	0.48
5	A/5	11.87
6	A/6	11.87
7	A/7	15.22
8	A/8	25.19
9	A/9	15.58
10	A/10	2.15
11	A/11	4.19
12	A/12	2.98
13	A/13	1.75
14	A/14	0.98

**Table 4:** The evaluation of the antimicrobial properties of isolated fractions.

Scheme	Antimicrobial Activity (Diameter of the Growth Inhibition Zone, mm)		
	I	II	III
A/1–A/4	14.0 ± 0.3 ^a^	12.0 ± 0.3 ^a^	16.0 ± 0.3 ^a^
A/5–A/7	16.0 ± 0.3 ^ac^	13.0 ± 0.2 ^a^	14.0 ± 0.2 ^a^
A/8–A/11	0.0 ± 0.0 ^b^	0.0 ± 0.0 ^b^	0.0 ± 0.0 ^b^
A/12–A/14	0.0 ± 0.0 ^b^	0.0 ± 0.0 ^b^	0.0 ± 0.0 ^b^
Ampicillin	19.0 ± 0.3 ^c^	15.0 ± 0.3 ^a^	−
Fluconazole	−	−	20.0 ± 0.3 ^c^

I—*B. cereus,* II—*E. coli*, III—*C. albicans*. The data are expressed as mean ± SE (*n* = 3). Values in columns followed by the same letter do not differ significantly (*p* < 0.05).

## Data Availability

Data are contained within the article.

## Supplementary Materials

The following are available online at https://www.mdpi.com/article/10.3390/antibiotics10080927/s1, Figure S1: Phylogenetic tree showing the relative position of isolates 1 (*Bacillus megaterium*) based on 16S rDNA sequences, using the neighbor-joining method. Figure S2: Phylogenetic tree showing the relative position of isolates 2 (*Pseudomonas fluorescens*) based on 16S rDNA sequences, using the neighbor-joining method. Figure S3: Phylogenetic tree showing the relative position of isolates 3 (*Pseudomonas putida*) based on 16S rDNA sequences, using the neighbor-joining method. Figure S4: Phylogenetic tree showing the relative position of isolates 4 (*Pseudomonas aeruginosa*) based on 16S rDNA sequences, using the neighbor-joining method. Figure S5: Phylogenetic tree showing the relative position of isolates 5 (*Micrococcus luteus*) based on 16S rDNA sequences, using the neighbor-joining method. Figure S6: Phylogenetic tree showing the relative position of isolates 6 (*Pseudomonas oleovorans*) based on 16S rDNA sequences, using the neighbor-joining method. Figure S7: Phylogenetic tree showing the relative position of isolates 7 (*Acinetobacter calcoaceticus*) based on 16S rDNA sequences, using the neighbor-joining method.

## Appendix A

**Table A1:** The Cultural and Morphological Properties of Isolates from the Bottom Sediments of Baikal Lake.

Samples of Isolates	Morphology of Colonies	Microscopic Characteristics
No. 1	Colonies are flat, rough, from off-white to yellow, with a 2 to 5 mm diameter.	Gram-positive spore-forming rods 0.5–1.5 µm in size with rounded ends. They are located singly or in pairs, sometimes they form chains. Cells are motile when using hanging drop method.
No. 2	Colonies are flat, convex, 1.5–3.0 mm in diameter, grayish.	Gram-negative rods, with polar flagella, non-spore-forming.Cells are motile when using hanging drop method.
No. 3	Colonies are round, flat, smooth, opaque, 2–3 mm in diameter, white, with uneven edges.	Gram-negative rods (straight or curved), do not form spores. Cells are motile when using hanging drop method.
No. 4	Colonies are convex, irregular form, beige.	Gram-negative rods (straight or slightly curved), do not form spores. Cells are motile when using hanging drop method.
No. 5	Colonies are round, shiny, smooth, opaque, yellow in color.	Gram-positive bacteria, spherical, arranged in pairs or in chains, do not form spores.
No. 6	Colonies are smooth, with smooth edges, convex, uniform consistency, 5 mm in diameter, light cream color.	Gram-negative rods. Cells are motile when using hanging drop method.
No. 7	Colonies are round, small, shiny, with a smooth edge, colorless.	Gram-negative thick, straight rods, arranged in pairs or chains, do not form spores and flagella.

## Appendix B. Sequences of the 18S Ribosomal RNA Gene of the *Bacillus megaterium*

GAGAGTTTGATCCTGGCTCAGGATNAACGCTGGCGGCGTGCCTAATACATGCAAGTNGAGCGAACTNATTAGAAGCTTGCTTNTATGACGTNAGCGGCGGACGGGTGAGTNACACGTGGGCAACCTGCCTGTAAGACTGGGATAACTTCGGGAAACCGAAGCTAATACCGGATAGGATCTTCTCCTTCATGGGAGATGATTGAAAGATGGTTTCGGCTATCACTTACAGATGGGCCCGCGGTGCATTAGCTNGTTGGTGAGGTAACGGCTCACCAAGGCAACGATGCATAGCCGACCTGAGAGGGTNATNGGCCACACTGGGACTGAGACACGGCCCAGACTCCTACGGGAGGCAGCAGTAGGGAATCTTCCGCAATGGACGAAAGTCTGACGGAGCAACGCCGCGTGAGTGATGAAGGCTNTCGGGTCGTAAAACTCTGTTGTTAGGGAAGAACAAGTACNAGAGTAACTNCTNGTNCCTTGACGGTACCTAACCAGAAAGCCACGGCTAACTACGTGCCAGCAGCCCCGGTNATACGTAGGTGGCAAGCGTNATCCGGAATTATTGGGCGTNAAGCGCGCGCAGGCGGTTTCTTAAGTCTNATGTGAAAGCCCACGGCTNAACCGTNGAGGGTCATTGGAAACTGGGGAACTNGAGTGCAGAAGAGAAAAGCGGAATTCCACGTGTAGCGGTNAAATGCGTAGAGATGTGGAGGAACACCAGTGGCGAAGGCGNCTNTNTNGTCTGTAACTGACGCTNAGGCGCGNAAGCGTGGGGAGCAAACAGGATTAGATACCCTGGTNGTCCACGCCGTAAACGATGAGTGCTAAGTGTTAGAGGGTTTCCGCCCTNTAGTGCTNCACTAACGCATNAAGCACTNCGCCTNGGGAGTACGGTCGCAAGACTNAAACTCAAAGGAATTGACGGGGGCCGCACAAGCGGTGGAGCATGTNGTTTAATTCGAAGNAACGCGAAGAACCTTACCAGGTCTNGACATCCTCTGACAACTCTAGAGATAGAGNGTTCCTTCGGGGNACAGAGTGACAGGTGGNGCATNGTNGTCGTCAGCTNGTGTCGTGAGATGTTGGGTNAAGTCCCGCAACGAGCGCAACCCTTGATCTTAGTTGCCAGCATTCAGTNGGGCACTCTAAGGTNACTGCCGGTGACAAACCGGAGGAAGGTGGGGATGACGTCAAATCATCATGCCCCTTATGACCTNGGCTACACACGTGCTACAATGGATGGTACAAAGGGCTGCAAGACCGCGAGGTNAAGCCAATCCCATNAAACCATNCTCAGTTCGGATNGTAGGCTGCAACTCGCCTNCATGAAGCTNGAATCGCTAGTAATCGCGGATCAGCATGCCGCGGTNAATACGTTCCCGGGCCTNGTACACACCGCNCGTCACACCACGAGAGTT

## Appendix C. Sequences of the 18S Ribosomal RNA Gene of the *Pseudomonas fluorescens*

AGTTTGATCCTGGCTCAGATTGAACGCTGGCGGCAGGCCTAACACATGCAAGTCGAGCGGTAGAGAGAAGCTTGCTTCTCTTGAGAGAGGCGGACGGGTGAGTAAAGCCTAGGAATCTGCCTGGTAGTGGGGGATAACGTTCGGAAACGGACGCTAATACCGCATACGTCCTACGGGAGAAAGCAGGGGACCTTCGGGCCTTGCGCTATCAGATGAGCCTAGGTCGGATTAGCTAGTTGGTGAGGTAATGGCTCACCAAGGCGACGATCCGTAACTGGTCTGAGAGGATGATCAGTCACACTGGAACTGAGACACGGTCCAGACTCCTTCGGGAGGCAGCAGTGGGGAATATTGGACAATGGGCGAAAGCCTGATCCAGCCATGCCGCGTGTGTTAAGAAGGTCTTCGGGTTGTAAAGCACTTTAAGTTGGGAGGAAGGGCATTAACCTAATACGTTAGTGTTTTGACGTTACCGACAGAATAAGCACCGGCTAACTCTGTGCCAGCAGCCGCGGTAATACAGAGGGTGCAAGCGTTAATCGGAATTACTGGGCGTAAAGCGCGCGTAGGTGGTTTGTTAAGTTGGATGTGAAATCCCCGGGCTCAACCTGGGAACTGCATTCAAAACTGACTGACTAGAGTATGGTAGAGGGTGGTGGAANTTCCTGTGTAGCGGTGAAATGCGCAGATATAGGAAGGAACACCAGTGGCGAAGGCGACCACCTGGACTAATACTGACACTGAGGTGCGAAAGCGTGGGGAGCAAACAGGATTAGATACCCTGGTAGTCCACGCCGTAAACGATGTCAACTAGCCGTTGGGAGCCTTGAGCTCTTAGTGGCGCAGCTAACGCATTAAGTTGACCGCCTGGGGAGTACGGCCGCAAGGTTAAAACTCAAATGAATTGACGGGGGCCCGCACAAGCGGTGGAGCATGTGGTTTAATTCGAAGCAACGCGAAGAGCCTTACCAGGCCTTGACATCCAATGAACTTTCTAGAGATAGATTGGTGCCTTCGGGAACATTGAGACAGGTGCTGCATGGCTGTCGTCAGCTCGTGTCGTGAGATGTTGGGTTAAGTCCCGTAACGAGCGCAACCCTTGTCCTTAGTTACCAGCACGTAATGGTGGGCACTCTAAGGAGACTGCCGGTGACAAACCGGAGGAAGGTGGGGATGACGTCAAGTCATCATGGCCCTTACGGCCTGGGCTACACACGTGCTACAATGGTCGGTACAGAGGGTTGCCAAGCCGCGAGGTGGAGCTAATCCCTCAAAACCGATCGTAGTCCGGATCGTAGTCTGCAACTCGACTGCGTGAAGTCGGAATCGCTAGTAATCGCGAATCAGAATGTCGCGGTGAATACGTTCCCGGGCCTTGTACACACCGCCCGTCACACCATGGGAGTGGGTTGCACCAGAAGTAGCTAGTCTAACCTTNCGGAGGGCGGTTACCACGGTGTGATTCATGACTGGGGTGAAGTCGTAACAAGGTAGCCGTAGGGGAACCTGCGGCTGGATCACCTCCTT

## Appendix D. Sequences of the 18S Ribosomal RNA Gene of the *Pseudomonas putida*

AGTTTGATCCTGGCTCAGATTGAACGCTGCCGGCAGGCCTAACACATGCAAGTCGAGCGGATGAGAAGAGCTTGCTCTTCGATTCAGCGGCGGACGGGTGCGTAATGCCTAGGAATCTGCCTGGTAGTGGGGGACAACGTTTCGAAAGGAACGCTAATACCGCATACGTCCTACGGGAGAAAGCGGGGGACCTTCGGGCCTTGCGCTATCAGATGAGCCTAGGTCGGATTAGCTAGTTGGTGAGGTAATGGCTCACCAAGGCGACGATCCGTAACTGGTCTGAGAGGATGATCAGTCACACTGGAACTNNGACACGGTCCAGACTCCTACGGGAGGCAGCAGTGGGGAATATTGGACAATGGGCGAAAGCCTGATCCAGCCATGCCGCGTGTGTTAAGAAGGTCTTCGGATTGTAAAGCACTTTAAGTTGGGAGGAAGGGCATTAACCTAATACGTTAGTGTTTTCACGTTACCGACAGAATAAGCACCGGCTAACTCTGTGCCAGCAGCCGCGGTAATACAGAGGGTGCAAGCGTTAATCGGAATTACTGGGCGTAAAGCGCGCGTGGGTGGTTTGTTAAGTTGGATGTGAAAGCCCCGGGCTCAACCTGGGAACTGCATCCAAAACTGGCAAGCTAGAGTACGGTAGAGGGTGGTGGAATTTCCTGTGTAGCGGTGAAATGCGTAGATATAGGAAGGAACACCAGTGGCGAAAGCGACCACCTGGACTGATACTGACACTGAGGTGCGAAAGCGTGGGGAGCAAACAGGATTAGATACCCTGGTAGTCCACGCCGTAAACGATGTCAACTAGCCGTTGGAATCCTTGAGATTTTAGTGGCGCAGCTAACGCATTAAGTTGACCGCCTGGGGAGTACGGCCGCAAGGTTAAAACTCAAATGAATTGACGGGGGCCCGCACAAGCGGTGGAGCATGTGGTTTAATTCGAAGCAACGCGAAGAACCTTACCAGGCCTTGACATGCAGAGAACTTTCCAGAGATGGATTGGTGCCTTCGGGAACTCTGACACAGGTGCTGCATGGCTGTCGTCAGCTCGTGTCGTGAGATGTTGGGTTAAGTCCCGTAACGAGCGCAACCCTTGTCCTTAGTTACCAGCACGTAATGGTGGGCACTCTAAGGAGACTGCCGGTGACAAACCGGAGGAAGGTGGGGATGACGTCAAGTCATCATGGCCCTTACGGCCTGGGCTACACACGTGCTACAATGGTCGGTACAGAGGGTTGCCAAGCCGCGAGGTGGAGCTAATCTCACAAAACCGAACGTAGTGCGGTTCGCAGTCTGCAACTCGACTGCGTGAAGTCGGAATCGCTAGTAATCGCGAATCAGAATGTCGCGGTGAATACGTTCCCGGGCCTTGTACACACCGCCCGTCACACCATGGGAGTGGGTTGCACCAGAAGTAGCTAGTCTAACCTTCGGGAGGANNGTTACCACGGTGTGATTCATGACTGGGGTGAAGTCNTAACAAGGTAGCCGTAGGGGAACCTGCGGCTGGATCACCTCCTT

## Appendix E. Sequences of the 18S Ribosomal RNA Gene of the *Pseudomonas aeruginosa*

GAACTGAAGAGTTTGATCATGGCCCAGATTGAACGCTGGCAGCAGGGGCCTTCAACACATGCAAGTCGAGCTTATGAAGGGAGCTTGCCTTGGATTCAGCGGCGGACGGGTGAGTAATGCCTAGGAATCTGCCTGGTAGTGGGGGATAACGTCCGGAAACGNNCGCTAATACCGCATACGTCCTGAGGGAGAAAGTCGGGGATCTTCGGACCTCACGCTATCAGATGAGCCTAGGTCGGATTAGCTAGTTGGTGGGGTAAAGGCCTACCAAGGCGACGATCCGTAACTGGTCTGAGAGGATGATCAGTCACACTGGAACTGAGACACGGTCCAGACTCCTACGGGAGGCAGCAGTGGGGAATATTGGACAATGGGCGCAAGNNTGATCCAGCCATGCCGCGTGTGTGAAGAAGGTCTTCGGATTGTAAAGCACTTTAAGTTGGGAGGAAGGGCAGTAAGTTAATACCTTGCTGTTTGACGTTACCAACAGAATAAGCACCGGCTAACTTCGTGCCAGCAGCCGCGGTAATACGAAGGGTGCAAGCGTTAATCGGAATTACTGGGCGTAAAGCGCGCGTAAGTGGTCCAGCAAGCTTGATGTGAAATCCCCGGGCTCAACCTGGGAACTGCATCCAAAAGCTACTGAGCTAGAGTACGGTAGAGGTGGTAGAATTTCCTGTGTAGCGGTGAAATGCGTAGATATAGGAAGGAACACCAGTGGCGAAGGCGACCACCTGGACTGTACTGACACTGAGGTGCGAAAGCGTGGGGAGCAAACAGGATTAGATACCCTGGTAGTCCACGCCGTAAACGATGTCGACTAGCCGTTGGGATCCTTGAGATCTTAGTGGCGCACGTAACGCGATAAGTCGACCGCCTGGGGAGTACGGCCGCAAGGTTAAAACTCAAATGAATTGACGGGGGCCCGCACAAGCGGTGGAGCATGTGGTTTAATTCGAAGCAACGCGAAGAACCTTACCTGGCCTTGACATGCTGAGAACTTTCCAGAGATGGATTGGTGCCTTCGGGAACAGAGACACAGGTGCTGCATGGCTGTCGTCAGCTCGTGTCGTGAGATGTTGGGTTAAGTCCCGTAACGAGCGCAACCCTTGTCCTTAGTTACCAGCACCTCGGGTGGGCACTCTAAGGAGACTGCCGGTGACAAACCGGAGGAAGGTGGGGATGACGTCAAGTCATCATGGCCCTTACGGCCAGGGCTACACACGTGCTACAATGGTCGGTACAAAGGGTTGCCAAGCCGCGAGTGGGAGCTAATCCCATAAAACCGATCGTAGTCCGGATCGCAGTCTGCAACTCGACTGCGTGAAGTCGGAATCGCTAGTAAACGTGAATCAGAATGTCACGGTGAATACGTCCCCGGGCCTTGTACACACCGCCCGTCACACCATGGGAGTGGGTTGCTCCAGTAGTAGCTAGTCTAACCGCAAGGGGGACGGTTACCACGGAGTGATTCATGACTGGGGTGAAGTCGTAACAAGGTAGCCGTAGGGGAACCTGCGGCTGGATCACCTCCTTA

## Appendix F. Sequences of the 18S Ribosomal RNA Gene of the *Micrococcus luteus*

GGCTCAGGATGATCGCTGGCGGCGTGCTTAACACATGCAAGTCGAACGATGAAGCCCAGCTTGCTGGGTGGATTAGTGGCGAACGGGTGAGTAACACGTGAGTAACCTGCCCTTAACTCTGGGATAAGCCTGGGAAACTGGGTCTAATACCGGATAGGAGCGTCCACCGNATGGTGGGTGTTGGAAAGATTTATCGGTTTTGGATGGACTCGCGGCCTATCAGCTTGTTGGTGAGGTAATNNCTCACCAAGGCGACGACGGGTAGCCGGCCTGAGAGGGTGACCGGCCACACTGGGACTGAGACACGGCCCAGACTCCTACGGGAGGCAGCAGTGGGGAATATTGCACAATGGGCGAAAGCCTGATGCAGCGACGCCGCGTGAGGGATGACGGCCTTCGGGTTGTAAACCTCTTTCAGTAGGGAAGAAGCGAAAGTGACGGTACCTGCAGAAGAAGCACCGGCTAACTACGTGCCAGCAGCCGCGGTAATACGTAGGGTGCGAGCGTTATCCGGAATTATTGGGCGTAAAGAGCTCGTAGGCGGTTAGTCGCGTCTGTCGTGAAAGTCCGGGGCTTAACCCCGGATCTGCGGTGGGTACGGGCAGACTAGAGTGCAGTAGGGGAGACTGGAATTCCTGGTGTAGCGGTGGAATGCGCAGATATCAGGAGGAACACCGATGGCGAAGGCAGGTCTCTGGGCTGTAACTGACGCTGAGGAGCGAAAGCATGGGGAGCGAACAGGATTAGATACCCTGGTAGTCCATGCCGTAAACGTTGGGCACTAGGTGTGGGGACCATTCCACGGTTTCCGCGCCGCAGCTAACGCATTAAGTGCCCCGCCTGGGGAGTACGGCCGCAAGGCTAAAACTCAAAGGAATTGACGGGGGCCCGCACAAGCGGCGGAGCATGCGGATTAAATCGATGCAACGCGAAGAACCTTACCAAGGCTTGACATGTTCCCGATCGCCGTAGAGATACGATTTCCCCTTTGGGGCGGGTTCACAGGTGGTGCATGGTTGTCGTCAGCTCGTGTCGTGAGATGTTGGGTTAAGTCCCGCAACGAGCGCAACCCTCGTNCCATGTTGCCAGCACGTAATGGTGGGGACTCATGGGAGACTGCCGGGGTCAACTCGGAGGAAGGTGAGGACGACGTCAAATCATCATGCCCCTTATGTCTTGGGCTTCACGCATGCTACAATGGCCGGTACAATGGGTTGCGATACTGTGAGGTGGAGCTAATCCCAAAAAGCCGGTCTCAGTTCGGATTGGGGTCTGCAACTCGACCCCATGAAGTCGGAGTCGCTAGTAATCGCANNTCAGCAACGCTGCGGTGAATACGTTCCCGGGCCTTGTACACACCGCCCGTCAAGTCACGAAAGTCGGTAACACCCGAAGCCGGTGGCCTAACCCTTGTGGGA

## Appendix G. Sequences of the 18S Ribosomal RNA Gene of the *Pseudomonas oleovorans*

ATTGAACGCTGGCGGCAGGCCTAACACATGCAAGTCGAGCGGATGAAGGGAGCTTGCTCCCTGATTTAGCGGCGGACGGGTGAGTAATGCCTAGGAATCTGCCTGGTAGTGGGGGATAACGTTCCGAAAGGAACGCTAATACCGCATACGTCCTACGGGAGAAAGCAGGGGACCTTCGGGCCTTGCGCTATCAGATGAGCCTAGGTCGGATTAGCTAGTTGGTGAGGTAATGGCTCACCAAGGCGACGATCCGTAACTGGTCTGAGAGGATGATCAGTCACACTGGAACTGAGACACGGTCCAGACTCCTACGGGAGGCAGCAGTGGGGAATATTGGACAATGGGCGAAAGCCTGATCCAGCCATGCCGCGTGTGTGAAGAAGGTCTTCGGATTGTAAAGCACTTTAAGTTGGGAGGAAGGGCAGTAAGCTAATACCTTGCTGTTTTGACGTTACCGACAGAATAAGCACCGGCTAACTTCGTGCCAGCAGCCGCGGTAATACGAAGGGTGCAAGCGTTAATCGGAATTACTGGGCGTAAAGCGCGCGTAGGTGGTTCGTTAAGTTGGATGTGAAAGCCCCGGGCTCAACCTGGGAACTGCATCCAAAACTGGCGAGCTAGAGTACGGTAGAGGGTGGTGGAATTTCCTGTGTAGCGGTGAAATGCGTAGATATAGGAAGGAACACCAGTGGCGAAGGCGACCACCTGGACTGATACTGACACTGAGGTGCGAAAGCGTGGGGAGCAAACAGGATTAGATACCCTGGTAGTCCACGCCGTAAACGATGTCAACTAGCCGTTGGGTTCCTTGAGAACTTAGTGGCGCAGCTAACGCATTAAGTTGACCGCCTGGGGAGTACGGCCGCAAGGTTAAAACTCAAATGAATTGACGGGGGCCCGCACAAGCGGTGGAGCATGTGGTTTAATTCGAAGCAACGCGAAGAACCTTACCTGGCCTTGACATGCTGAGAACTTTCCAGAGATGGATTGGTGCCTTCGGGAACTCAGACACAGGTGCTGCATGGCTGTCGTCAGCTCGTGTCGTGAGATGTTGGGTTAAGTCCCGTAACGAGCGCAACCCTTGTCCTTAGTTACCAGCACGTAATGGTGGGCACTCTAAGGAGACTGCCGGTGACAAACCGGAGGAAGGTGGGGATGACGTCAAGTCATCATGGCCCTTACGGCCAGGGCTACACACGTGCTACAATGGTCGGTACAAAGGGTTGCCAAGCCGCGAGGTGGAGCTAATCCCATAAAACCGATCGTAGTCCGGATCGCAGTCTGCAACTCGACTGCGTGAAGTCGGAATCGCTAGTAATCGTGAATCAGAATGTCACGGTGAATACGTTCCCGGGCCTTGTACACACCGCCCGTCACACCATGGGAGTGGGTTGCTCCAGAAGTAGCTAGTCTAACCTTCGGGGGGACGGTTACCACGGAGTGATTCATGACTGGGGTGAAGTCGTAACAAGGTAGCCGTAGGGGAACCTGC

## Appendix H. Sequences of the 18S Ribosomal RNA Gene of the *Acinetobacter calcoaceticus*

TCCTGGCTCAGATTGAACGCTGGCGGCAGGCTTAACACATGGAAGTCGAGCGGAGAGAGGTAGCTTGCTACTGATCTTAGCNNCGGACGGGTGAGTAATGCTTAGGAATCTGCCTATTAGTGGGGGACAACATTTCGAAAGGAATGCTAATACCGCATACGTCCTACGGGAGAAAGCAGGGGATCTTCGGACCTTGCGCTAATAGATGAGCCTAAGTCGGATTAGCTAGTTGGTGGGGTAAAGGCCTACCAAGGCGACGATCTGTAGCGGGTCTGAGAGGATGATCCGCCACACTGGGACTGAGACACGGCCCAGACTCCTACGGGAGGCAGCAGTGGGGAATATTGGACAATGGGCGCAAGCCTGATCCAGCCATGCCGCGTGTGTGAAGAAGGCCTTATGGTTGTAAAGCACTTTAAGCGAGGAGGAGGCTACTTTAGTTAATACCTAGAGATAGTGGACGTTACTCGCAGAATAAGCACCGGCTAACTCTGTGCCAGCAGCCGCGGTAATACAGAGGGTGCAAGCGTTAATCGGATTTACTGGGCGTAAAGCGCGCGTAGGCGGCTAATTAAGTCAAATGTGAAATCCCCGAGCTTAACTTGGGAATTGCATTCGATACTGGTTAGCTAGAGTGTGGGAGAGGATGGTAGAATTCCAGGTGTAGCGGTGAAATGCGTAGAGATCTGGAGGAATACCGATGGCGAAGGCAGCCATCTGGCCTAACACTGACGCTGAGGTGCGAAAGCATGGGGAGCAAACAGGATTAGATACCCTGGTAGTCCATGCCGTAAACGATGTCTACTAGCCGTTGGGGCCTTTGAGGCTTTAGTGGCGCAGCTAACGCGATAAGTAGACCGCCTGGGGAGTACGGTCGCAAGACTAAAACTCAAATGAATTGACGGGGGCCCGCACAAGCGGTGGAGCATGTGGTTTAATTCGATGCAACGCGAAGAACCTTACCTGGCCTTGACATAGTAAGAAGTTTCCAGAGATGGATTGGTGCCTTCGGGAACTTACATACAGGTGCTGCATGGCTGTCGTCAGCTCGTGTCGTGAGATGTTGGGTTAAGTCCCGCAACGAGCGCAACCCTTTTCCTTATTTGCCAGCGAGTAATGTCGGGAACTTTAAGGATACTGCCAGTGACAAANTGGAGGAAGGCGGGGACGACGTCAAGTCATCATGGCCCTTACGGCCAGGGCTACACACGTGCTACAATGGTCGGTACAAAGGGTTGCTACCTAGCGATAGGATGCTAATCTCAAAAAGCCGATCGTAGTCCGGATTGGAGTCTGCAACTCGACTCCATGAAGTCGGAATCGCTAGTAATCGCGGATCAGAATGCCGCGGTGAATACGTTCCCGGGCCTTGTACAGACCGCCCGTCACACCATGGGAGTTTGTTGCACCAGAAGTAGCTAGCCTAACTGCAAAGAGGGCGGTTACCACGGTGTGGCCGATGACTGGGGTGAAGTCGTAACAAGGTAGCCGTAGGGGAACCTGCGGCTGGATCACC

## Appendix I

**Table A2:** Amino Acid Sequences of Peptides Produced by Selected Microorganisms.

(1)	TrpAspAsnAspThrGlMetAspAspAlaAspGlyAspSerGlyGlyThrAlaGly
(2)	TrpAspAsnAspThrGlMetAspAsnAlaAspSerAspSerGlyGlyThrAlaGly
(3)	PhenAspAlaAspThrGlMetAspAspAlaTrpGlyAspSerGlyMetThrAlaGly

## References

[1] Arzhannikov S.G., Ivanov A.V., Arzhannikova A.V., Demonterova E.I., Jansen J.D., Preusser F., Kamenetsky V.S., Kamenetsky M.B. (2018). Catastrophic events in the Quaternary out flow history of Lake Baikal. Earth Sci. Rev..

[2] Maksimenko S.Y., Zemskaya T.I., Pavlova O.N., Ivanov V.G., Buryukhaev S.P. (2008). Microbial community of the water column of the Selenga River-Lake Baikal biogeochemical barrier. Microbiology.

[3] Lew S., Glińska-Lewczuk K., Burandt P., Obolewski K., Goździejewska A., Lew M., Dunalska J. (2016). Impact of environmental factors on bacterial communities in floodplain lakes differed by hydrological connectivity. Limnologica.

[4] Bychkov I.V., Gagarinova O.V., Orlova I.I., Bogdanov V.N. (2018). Water Protection Zoning as an Instrument of Preservation for Lake Baikal. Water.

[5] Suslova M.Y., Grebenshchikova V.I. (2020). Water quality monitoring of the Angara River source. Limnol. Freshw. Biol..

[6] Shevchenko M.A., Suhih A.S., Bulgakova O.M. (2019). Analysis of psychrophilic microbiota of lake Baikal sediments. The Strategies of Modern Science Development, Proceedings of the XVII International Scientific-Practical Conference, Warsaw, Poland, 25 September 2019.

[7] Valverde A., Tuffin M., Cowan D.A. (2012). Biogeography of bacterial communities in hot springs: A focus on the actinobacteria. Extremophiles.

[8] Monciardini P., Iorio M., Maffioli S., Sosio M., Donadio S. (2014). Discovering new bioactive molecules from microbial sources. Microb. Biotechnol..

[9] Axenov-Gribanov D., Rebets Y., Tokovenko B., Voytsekhovskaya I., Timofeyev M., Luzhetskyy A. (2016). The isolation and characterization of actinobacteria from dominant benthic macroinvertebrates endemic to Lake Baikal. Folia Microbiol..

[10] Brunati M., Rojas J.L., Sponga F., Ciciliato I., Losi D., Göttlich E., Hoogd S., Genilloud O., Marinelli F. (2009). Diversity and pharmaceutical screening of fungi from benthic mats of Antarctic lakes. Mar. Genom..

[11] Imoff J.F., Labes A., Wise J. (2011). Bio-mining the microbial treasures of the ocean: New natural products. Biotechnol. Adv..

[12] Zhang L., Jungblut A.D., Hawes I., Andersen D.T., Sumner D.Y., Mackey T.J. (2015). Cyanobacterial diversity in benthic mats of the McMurdo Dry Valley lakes, Antarctica. Polar Biol..

[13] Galach’yants A.D., Bel’kova N.L., Sukhanova E.V., Romanovskaya V.A., Gladka G.V., Bedoshvili E.D., Parfenova V.V. (2016). Diversity and Physiological and Biochemical Properties of Heterotrophic Bacteria Isolated from Lake Baikal Neuston. Mikrobiologiia.

[14] Zimina M., Babich O., Prosekov A., Sukhikh S., Ivanova S., Shevchenko M., Noskova S. (2020). Overview of global trends in classification, methods of preparation and application of bacteriocins. Antibiotics.

[15] Cabello-Yeves P.J., Zemskaya T.I., Zakharenko A.S., Sakirko M.V., Ivanov V.G., Ghai R., Rodriguez-Valera F. (2019). Microbiome of the deep Lake Baikal, a unique oxic bathypelagic habitat. Limnol. Oceanogr..

[16] Belkova N., Parfenova V.V., Kostornova T.Y., Denisova L.Y., Zaĭchikov E.F. (2003). Microbial Biodiversity in the Water of Lake Baikal. Microbiology.

[17] Zemskaya T., Cabello-Yeves P.J., Pavlova O., Rodriguez-Valera F. (2020). Microorganisms of Lake Baikal—the deepest and most ancient lake on Earth. Appl. Microbiol. Biotechnol..

[18] Strohl W.R., Strohl W.R. (1997). Industrial antibiotics: Today and the future. Biotechnology of Antibiotics.

[19] Sherbakov Y. (1999). Molecular phylogenetic studies on the origin of biodiversity in Lake Baikal. Trends Ecol. Evol..

[20] Terkina I.A., Drukker V.V., Parfenova V.V., Kostornova T.Y. (2002). The biodiversity of actinomycetes in Lake Baikal. Microbiology.

[21] Parfenova V.V., Gladkikh A.S., Belykh O.I. (2013). Comparative analysis of biodiversity in the planktonic and biofilm bacterial communities in Lake Baikal. Microbiology.

[22] Pavlova O.N., Zemskaya T.I., Gorshkov A.G., Kostornova T.Y., Khlystov O.M., Parfenova V. (2008). V Comparative characterization of microbial communities in two regions of natural oil seepage in Lake Baikal. Biol. Bull..

[23] Parfenova V.V., Pavlova O.N., Kravchenko O.S., Tulupova Y.R., Kostornova T.Y. (2010). investigation of distribution, species composition, and degree of resistance to antibiotics of the bacteria of the *Enterococcus* genus in Lake Baikal. Contemp. Probl. Ecol..

[24] Lomakina A.V., Pavlova O.N., Shubenkova O.V., Zemskaya T.I. (2009). Diversity of cultured aerobic organisms in the areas of natural oil seepage on Lake Baikal. Biol. Bull..

[25] Pavlova O.N., Bukin S.V., Lomakina A.V., Kalmychkov G.V., Ivanov V.G., Morozov I.V., Pogodaeva T.V., Pimenov N.V., Zemskaya T.I. (2014). Production of gaseous hydrocarbons by microbial communities of Lake Baikal bottom sediments. Microbiology.

[26] Bel’kova N.L., Dzyuba E.V., Klimenko E.S., Khanaev I.V., Denikina N.N. (2018). Detection and Genetic Characterization of Bacteria of the Genus Pseudomonas from Microbial Communities of Lake Baikal. Russ. J. Genet..

[27] Rojas J.L., Martín J., Tormoa J.R., Vicente F., Brunati M., Ciciliato I., Losi D., Van Trappen S., Mergaert J., Swings J. (2009). Bacterial diversity from benthic mats of Antarctic lakes as a source of new bioactive metabolites. Mar. Genom..

[28] Holt J.G., Krieg N.R., Sheath P.H.A., Stanley J.T., Williams S.T. (1994). Bergey’s Manual of Determinative Bacteriology.

[29] Straškrábová V., Izmest’yeva L.R., Maksimova E.A., Fietzd S., Nedoma J., Boroveca J., Kobanova G.I., Shchetinina E.V., Pislegina E.V. (2005). Primary production and microbial activity in the euphotic zone of Lake Baikal (Southern Basin) during late winter. Glob. Planet. Chang..

[30] Tosi S., Casado B., Gerdol R., Caretta G. (2002). Fungi isolated from Antarctic mosses. Polar Biol..

[31] Biondi N., Tredici M.R., Taton A., Wilmotte A., Hodgson D.A., Losi D., Marinelli F. (2008). Cyanobacteria from benthic mats of Antarctic lakes as a source of new bioactivities. J. Appl. Microbiol..

[32] Sorokin D.Y., Zhilina T.N., Lysenko A.M., Tourova T.P., Spiridonova E.M. (2006). Metabolic versatility of haloalkaliphilic bacteria from soda lakes belonging to the Alkalispirillum-Alkalilimnicola group. Extremophiles.

[33] Shillinger U., Lücke F.K. (1989). Antibacterial activity of Lactobacillus sake isolated from meat. J. Appl. Microbiol..

[34] Akasha I.A.M. (2014). Extraction and Characterisation of Protein Fraction from Date Palm (*Phoenix dactylifera* L.). Ph.D. Thesis.

[35] Bauer A.W., Kirby W.M.M., Sherris J.C., Turck M. (1966). Antibiotic susceptibility testing by a standardized single disk method. Am. J. Clin. Pathol..

[36] Van Trappen S., Mergaert J., Van Eygen S., Dawyndt P., Cnockaert M.C., Swings J. (2002). Diversity of 746 heterotrophic bacteria isolated from microbial mats from ten Antarctic lakes. Syst. Appl. Microbiol..

[37] Kimura H., Sashihara T., Matsusaki H., Sonomoto K., Ishizaki A. (1998). Novel bacteriocins of Pediococcus sp. ISK-1 isolated from well-aged bed of fermented rice bran. Ann. N. Y. Acad. Sci..

