# Supplementary material for: Antimicrobial Potential of Microorganisms Isolated from the Bottom Sediments of Lake Baikal
Source: Antibiotics (Basel). 2021 Jul 30;10(8):927. doi: 10.3390/antibiotics10080927 (PMC8388859; doi:10.3390/antibiotics10080927)
Supplement: Supplementary file 1 [file antibiotics-10-00927-s001.zip › antibiotics-1267748-Supplementary.pdf]

antibiotics

MDPI

Article

Antimicrobial Potential of Microorganisms Isolated from the Bottom Sediments of Lake Baikal

Olga Babich [1], Margarita Shevchenko [1],Svetlana Ivanova [2,3,*],Valery Pavsky [3], Maria Zimina [1], Svetlana Noskova [1], Veronika Anohova [1], Evgeny Chupakhin [1] and Stanislav Sukhikh [1]

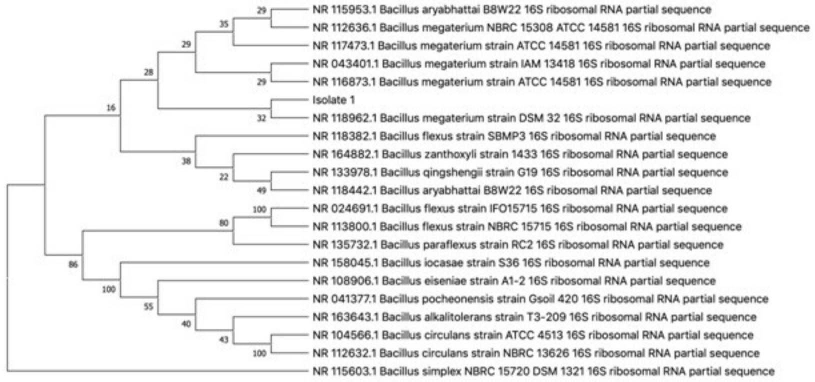

Figure S1. Phylogenetic tree showing the relative position of isolates 1 (*Bacillus megaterium*) based on 16S rDNA sequences, using the neighbor-joining method.

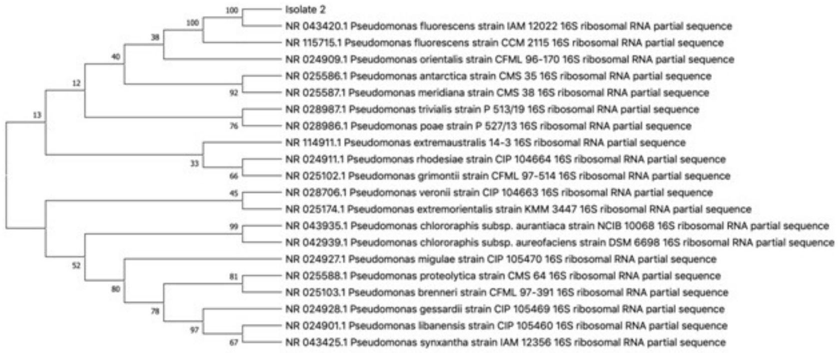

Figure S2**:** Phylogenetic tree showing the relative position of isolates 2 (*Pseudomonas fluorescens*) based on 16S rDNA sequences, using the neighbor-joining method.

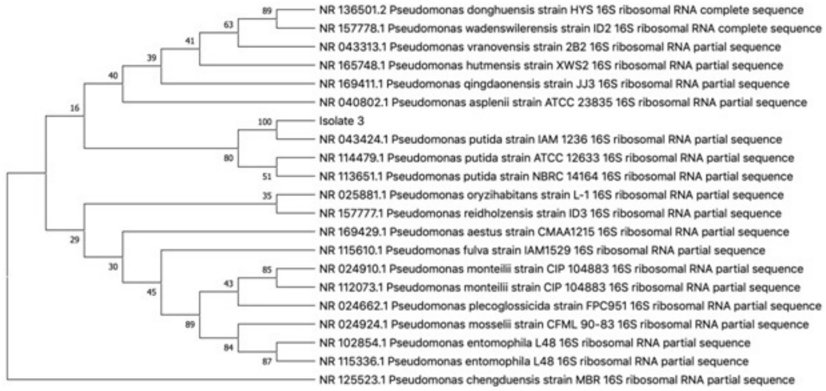

Figure S3: Phylogenetic tree showing the relative position of isolates 3 (*Pseudomonas putida*) based on 16S rDNA sequences, using the neighbor-joining method.

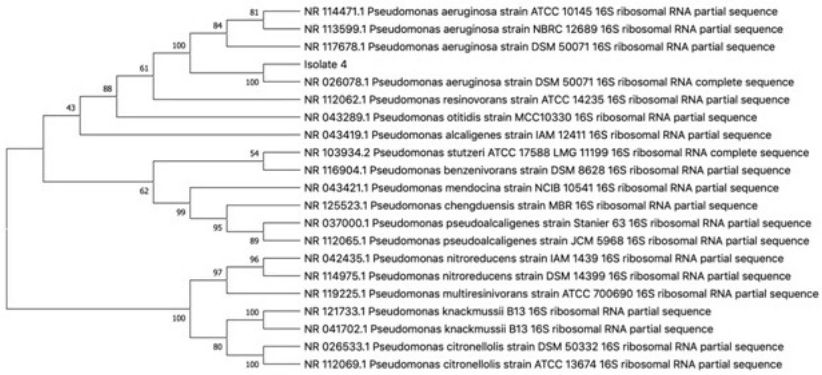

Figure S4: Phylogenetic tree showing the relative position of isolates 4 (*Pseudomonas aeruginosa*) based on 16S rDNA sequences, using the neighbor-joining method.

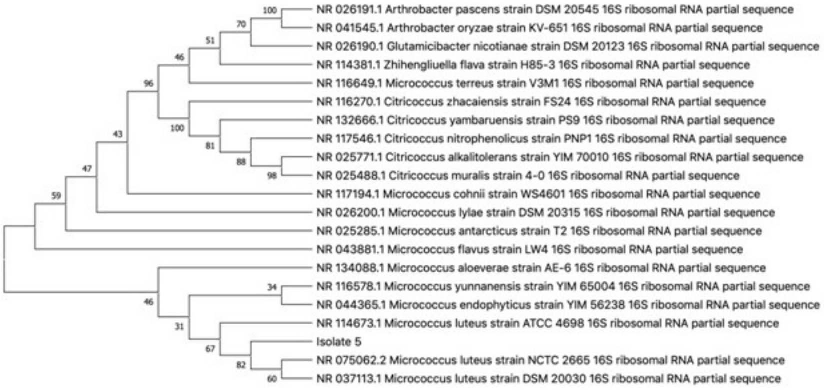

Figure S5: Phylogenetic tree showing the relative position of isolates 5 (*Micrococcus luteus*) based on 16S rDNA sequences, using the neighbor-joining method.

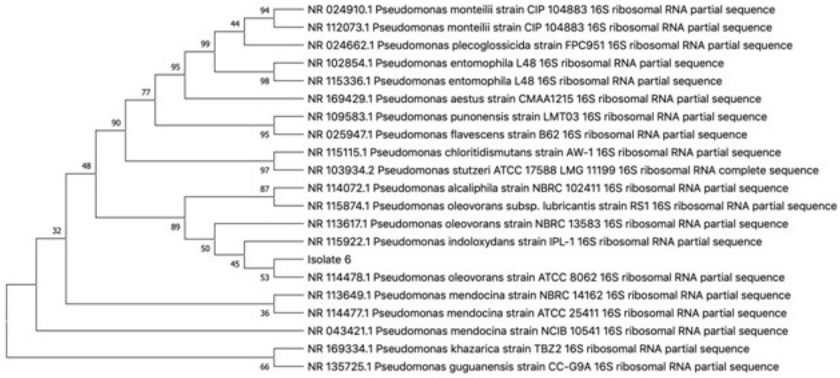

Figure S6: Phylogenetic tree showing the relative position of isolates 6 (*Pseudomonas oleovorans*) based on 16S rDNA sequences, using the neighbor-joining method.

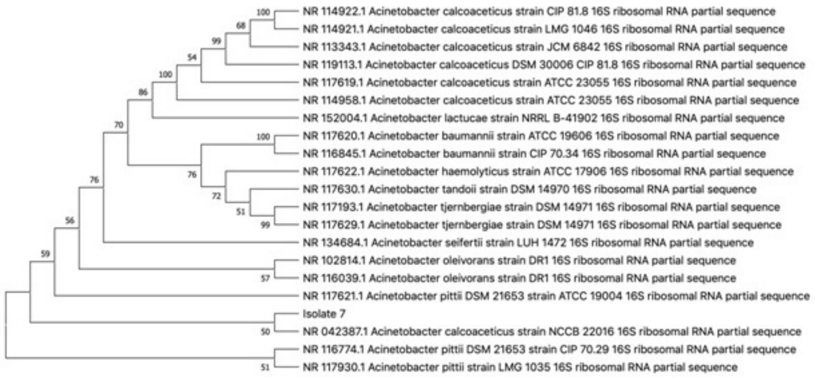

Figure S7: Phylogenetic tree showing the relative position of isolates 7 (*Acinetobacter calcoaceticus*) based on 16S rDNA sequences, using the neighbor-joining method.
